# Dissociation of categorical and coordinate spatial relations on dynamic network organization states

**DOI:** 10.3389/fnhum.2022.972375

**Published:** 2022-11-17

**Authors:** Xin Hao, Zhencai Chen, Taicheng Huang, Yiying Song, Xiangzhen Kong, Jia Liu

**Affiliations:** ^1^Key Laboratory of Adolescent Cyberpsychology and Behavior (CCNU), Ministry of Education, Wuhan, China; ^2^School of Psychology, Central China Normal University, Wuhan, China; ^3^Department of Psychology, Jiangxi University of Chinese Medicine, Nanchang, China; ^4^Department of Psychology, Tsinghua University, Beijing, China; ^5^Beijing Key Laboratory of Applied Experimental Psychology, Faculty of Psychology, Beijing Normal University, Beijing, China; ^6^Department of Psychology and Behavioral Sciences, Zhejiang University, Hangzhou, China

**Keywords:** categorical spatial relation, coordinate spatial relations, network organization, functional connectivity, dynamics

## Abstract

Humans can flexibly represent both categorical and coordinate spatial relations. Previous research has mainly focused on hemisphere lateralization in representing these two types of spatial relations, but little is known about how distinct network organization states support representations of the two. Here we used dynamic resting-state functional connectivity (FC) to explore this question. To do this, we separated a meta-identified navigation network into a ventral and two other subnetworks. We revealed a Weak State and a Strong State within the ventral subnetwork and a Negative State and a Positive State between the ventral and other subnetworks. Further, we found the Weak State (i.e., weak but positive FC) within the ventral subnetwork was related to the ability of categorical relation recognition, suggesting that the representation of categorical spatial relations was related to weak integration among focal regions in the navigation network. In contrast, the Negative State (i.e., negative FC) between the ventral and other subnetworks was associated with the ability of coordinate relation processing, suggesting that the representation of coordinate spatial relations may require competitive interactions among widely distributed regions. In sum, our study provides the first empirical evidence revealing different focal and distributed organizations of the navigation network in representing different types of spatial information.

## Introduction

Understanding spatial relations is hard for artificial intelligence, but easy for human being. Spatial relationships are critical to the formation of cognitive maps in navigation. We can use flexible representations to encode spatial relations either categorically, such that concerns the spatial layout formed by relative positions of objects (e.g., a house located in the left of the oak); or as coordinates, such that refers to the spatial locations in terms of metric units (e.g., a house located 2.5 m from the oak) (Kosslyn, 1987). The categorical spatial relationships capture abstract or general relations among object, such as “under”; by contrast, the coordinate spatial relationships reflect the exact metric or precise distance between objects. As evidenced in other domains, such as number (Feigenson et al., 2004; Pica et al., 2004) and color (Kay and Regier, 2003), there are profound distinctions between categorical and coordinate representations (Holden et al., 2010). Do the representations of categorical and coordinate spatial relations have distinct underlying neural substrates? Large majority of relevant neuroimaging studies concentrated on discovering the hemispheric lateralization dissociation between the two types of spatial relation representations in the prefrontal or parietal cortex, which based on making very basic task designs by using lines, crosses, or dots (Kosslyn et al., 1989; Trojano et al., 2002; Slotnick and Moo, 2006; van der Ham et al., 2009, 2013). However, a fundamental question remaining unclear is how distinct neural substrates in the large-scale navigational system underpinned the categorical and coordinate spatial relations.

Until now less attention has been paid to direct comparison between categorical and coordinate spatial relations representation under navigational system, and mix results are reported. The only known studies identify greater activation in the parietal cortex in the categorical condition, and higher activation in the medial temporal lobe (MTL) and dorsal striatum in the coordinate condition during spatial navigation (Baumann et al., 2012; Baumann and Mattingley, 2014); however, another study finds that the anterior temporal gyrus processes the categorical spatial information, and the left angular and inferior frontal gyrus processes more coordinate spatial information (Amorapanth et al., 2010). Importantly, neuropsychological studies provide some insight about some brain regions are conjointly and others distinctly recruited in the categorical and coordinate spatial relations. Within the scope of categorical/coordinate distinction, these shared and distinct activation regions mainly restrict to a small number of navigation-related regions of interest. On the one hand, the MTL has been found to play a central role in representing both categorical and coordinate spatial relations. For categorical spatial relations, patients with hippocampal damage are impaired in recognizing the relative relations among mountains (Hartley et al., 2007; Urgolites et al., 2017), and bilateral posterior hippocampus (HIP) are activated more highly for correct than incorrect recognition of ordinal location relations (Hannula and Ranganath, 2008). In addition, a recent study demonstrates greater activation in the left parahippocampal gyrus and retrosplenial cortex for processing of spatial relations than locations (Blacker and Courtney, 2016). Similarly, for coordinate spatial relations, the activation in either anterior or posterior HIP has been found correlated with the Euclidean distance (Evensmoen et al., 2013; Sherrill et al., 2013; Howard et al., 2014) path distance (Howard et al., 2014) and environment size (Baumann and Mattingley, 2013), and closer locations/items have higher neural pattern similarity in the HIP (Nielson et al., 2015; Deuker et al., 2016). On the other hand, compared with categorical spatial relations, more distributed regions beyond the MTL have been demonstrated to represent the coordinate spatial relations. For example, the medial prefrontal and medial posterior parietal regions show increased activation with closer distance to the goal (Spiers and Maguire, 2007; Viard et al., 2011), and the PCUN, insula, and anterior cingulate cortex show higher activation with further distance to the goal (Viard et al., 2011). In addition, distance-related adaptation effect is observed in the left inferior insula, anterior superior temporal sulcus, and right inferior temporal sulcus (Morgan et al., 2011). Taken together, existing evidence seems to suggest that the representation of categorical spatial relations mainly converges on the MTL, while the representation of coordinate spatial relations may also involve other spatial-related regions beyond the MTL (Ekstrom and Yonelinas, 2020).

Notably, it is increasingly recognized that different functions of the MTL may arise from its distinct intrinsic connectivity profiles with diverse cortical regions (Mahon and Caramazza, 2011; Sormaz et al., 2017). Yet, limited studies investigated the neural differences based on the intrinsic functional connectivity (FC) nature of the spatial relations. One suggestive study shows higher resting-state FC between the HIP and lingual gyrus is related to better ordinal relations memory (Sormaz et al., 2017). However, it remains unclear how different intrinsic connectivity patterns among the MTL and other spatial-related regions support representations of categorical and coordinate spatial relations in spatial navigation, respectively. Based on previous findings, we hypothesize that (1) the representation of categorical spatial relations would be mainly related to interactions within the MTL regions and (2) the representation of coordinate spatial relations would be related to more distributed interactions among navigation-related regions.

To test these two hypotheses, we used the meta-identified navigation network across the brain with the Neurosynth (Yarkoni et al., 2011), and decomposed it into a ventral subnetwork containing the MTL and other two subnetworks with a modularity analysis (Hao et al., 2016; Kong et al., 2016). Then, we characterized the intrinsic FC within the ventral subnetwork and that between the ventral and other subnetworks. Recent studies adopting dynamic FC approach have unveiled the time-varying nature of resting-state FC, indicating some degree of multi-stability for dynamic FC with multiple typical network states recurring during resting-state (Hutchison et al., 2013; Allen et al., 2014). The recurring FC states may manifest endogenous neural dynamics that are believed to underlie the flexibility of cognition and behavior, with different FC states relating to different cognitive functions (Shine et al., 2016). That is, representation of categorical and coordinate spatial relations may be associated with different dynamic FC states of the navigation network. Therefore, we calculated the FC matrices of all sliding time windows during resting-state and clustered them into typical dynamic FC states within the ventral subnetwork and between the ventral and other subnetworks, respectively. After MRI scanning, we used an ordinal scene recognition task to measure representation of categorical spatial relations, and distance test to measure representation of coordinate spatial relations. Finally, we correlated properties of the typical dynamic FC states with behavioral performances in the two tasks to examine whether and how distinct dynamic network states were associated with representations of categorical and coordinate spatial relations.

## Materials and methods

### Participants

Two hundred and twenty-six students (age range: 19–24; mean age = 21.66, *SD* = 1.00 years, 108 males) were recruited from Beijing Normal University, Beijing, China to participate in this study. This sample size is comparable with previous work (Wang et al., 2016; Hao et al., 2021) and exceeds prior fMRI studies in most cases. None of the participants reported a history of neurological or psychiatric disorders. This study is part of an ongoing project (Gene Environment Brain and Behavior) (Kong et al., 2017; Zhen et al., 2017). All experiments were in accordance with the ethical standards of the Institutional Review Board of Beijing Normal University and written informed consent was obtained from each participant before the experiment. One participant was excluded due to more than 0.2 mm in mean framewise displacements of the head motion.

### Behavior tasks

The behavior tasks were tested outside of the MRI scanner in a separate behavioral session, after the participants underwent MRI scanning.

### Assessment of categorical spatial relations

**FIGURE 1:**
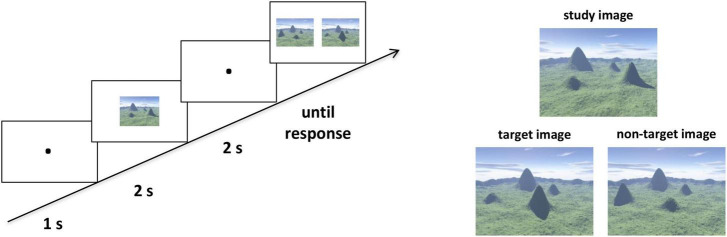
The ordinal scene recognition task to assess categorical spatial relations. **Left panel:** Example of a single trial. The participants were asked to identify the target image as quickly and accurately as possible in the test phase. **Right panel:** Examples of the study and test images. Two images were presented in the test phase, one of which was the target image and the other was the non-target image. The target image preserved all ordinal information from the study image but with a novel viewpoint. For the non-target image, the locations of two mountains were exchanged to the study image.

We assessed the participants’ ability to represent categorical spatial relations with an ordinal scene recognition task adapted from Hartley et al. (2007). The stimuli were all computer-generated landscapes, with four mountains varying in shape and size surrounded by a distant semicircular mountain range (Figure 1). There were 20 trials which were randomly mixed in the task. In the study phase of each trial, participants were required to study an image at the center of the screen for 2 s. Then after a delay of 2 s with fixation, two images from a novel viewpoint were presented in the test phase, including one target image which preserved all topographic information from the study image and one non-target image with the locations of two mountains exchanged with each other. The location of the target image on the left or right was randomized. The participants were asked to identify the target image as quickly and accurately as possible, by pressing “F” when the target image was on the left and “J” when the target image was on the right. Critically, the categorical spatial position among the four mountains extracted from the study images, which were interrupted in the non-target images. Then, we averaged the reaction times (RTs) of the correct trials for each participant. For visualization and enhancing readability, we converted these RTs into speed values using the classic formula: speed*_*i*_* = (Max–RT*_*i*_*)/(Max–Min), where *i* refer to each participant, and Max and Min correspond to the maximum and minimum RT across all participants (Hao et al., 2017). The speed values were used as the ordinal scene recognition scores.

### Assessment of coordinate spatial relations

According to the Lopez et al. (2020), we assessed the ability to represent coordinate spatial relations with the distance test. For the most noticeable way to assess the mentally represented distances is to compare the landmarks with each other (Lopez et al., 2020). The participant was presented with two well-known landmarks (i.e., buildings or statues) in the Beijing Normal University campus and required to choose the one closer to the building where the experiment was conducted (i.e., the starting point). It’s reasonable that coordinate judgments were made when representing distances metrically. Specifically, we used two surveys, which measured both the optimal path distance and straight-line Euclidean distance. For the optimal path distance survey, the participant was asked to select the optimal routes and avoid dead ends to assess the distances between the starting point and the landmarks; while in the Euclidean distance survey, the participant was asked to estimate the straight-line distances between the starting point and the landmarks. Each survey contained nine items, including eighteen prominent landmarks. Before the formal test, participants were provided with two practice items to familiarize with the task and were given feedback on each item. No feedback was provided in the formal test. We averaged the number of correct items of the path distance and Euclidean distance surveys as the distance score for each participant.

All participants had lived in the campus for more than 2 years at the time of the test. To ensure that all participants were familiar with the landmarks, they underwent a familiarity testing, in which they rated their degree of familiarity with each landmark on a scale ranging from one “very unfamiliar” to seven “very familiar.” The high mean score (6.10 ± 0.70) indicates relatively very familiar to all landmarks for each participant.

### Image acquisition and preprocessing

MRI scanning was conducted on a Siemens 3T scanner (MAGENTOM Trio, a Tim system) with a 12-channel phased-array head coil at Beijing Normal University Imaging Center for Brain Research, Beijing, China. T2*-weighted functional images in resting state were acquired using a gradient-echo, echo-planar imaging (EPI) sequence (TR = 2 s, TE = 30 ms, FA = 90°, number of slices = 33, voxel size = 3.125 mm × 3.125 mm × 3.6 mm, number of volumes = 240). During resting state scans, the participants were instructed to close their eyes, keep still, remain awake, and not think about anything systematically. Of note, each participant was asked whether he/she had fallen asleep during the scanning when the scan was finished. Those who reported having fallen asleep were required to rescan the resting-state imaging. In addition, high-resolution T1-weighted structure images were acquired using a magnetization-prepared rapid gradient-echo (MPRAGE) sequence (TR/TE/TI = 2,530/3.39/1,100 ms, FA = 7°, matrix = 256 × 256, voxel size = 1 mm × 1 mm × 1.33 mm, number of volumes = 128) for each participant.

For each participant, image preprocessing was conducted using FSL (FMRIB software Library^1^). Steps included the removal of the first four volumes for image stabilization, head motion correction (by aligning each volume to the middle volume of the image with MCFLIRT), spatial smoothing (with a Gaussian kernel of 6 mm full-width at half-maximum), grand-mean intensity normalization, and the removal of linear trend. Next, a band-pass temporal filter (0.01–0.1 Hz) was applied to reduce low-frequency drifts and high-frequency noise. Then, the physiological noise (such as cardiac and respiratory cycles), and nuisance signals from the white matter, global gray matter average, cerebrospinal fluid, six head motion correction parameters, and first derivatives of these signals were regressed out (Fox et al., 2005; Biswal et al., 2010). The residual time series obtained were registered to the MNI standard space with using FLIRT and then used for the dynamic FC analyses. The strength of the intrinsic FC between two regions was estimated using the Pearson’s correlation coefficients of the residual rs-fMRI time series.

### Functional organization of the navigation network

Considering the complexity of navigation system, we used a meta-analysis approach named Neurosynth (Yarkoni et al., 2011), to identify 23 highly navigation-relevant regions involving in navigation (Hao et al., 2021). After the preprocessing, we first computed the Fisher *z*-transformed Pearson correlation coefficients between the residual time series of each pair of the defined regions for each participant. To detect how interconnected regions formed functionally module structure in the navigation network, we conducted a modularity analysis on the averaged correlation matrix across participants. Specifically, we used the community Louvain algorithm in the brain connectivity toolbox (version 2017-01-15, Rubinov and Sporns, 2010). For the Louvain algorithm, we choose the default resolution parameter gamma = 1 and executed it in the MATLAB. Considering the conceptual advantages of unequal importance of positive and negative weights, we adopted an asymmetric modularity measure to avoid biased thresholding of the networks. Then, we ran the algorithm 1,000 times and obtained the auto-generated optimal community structure with a module partition number of three. Further spatial examination found that one of the modules contained most of well-established navigational specific regions in the MTL (e.g., the hippocampal formation and retrosplenial complex) (Ekstrom et al., 2017; Qiu et al., 2019), which was labeled as the ventral subnetwork. The other two modules contained a set of general cognitive regions in frontal and parietal cortex (e.g., the inferior parietal lobe and middle frontal gyrus).

### Dynamic functional connectivity state clustering and statistics

We characterized the dynamic FC by using sliding time-window correlation among the identified regions in navigation network. For each participant we constructed a tapered window by convolving a rectangular window (width = 50 s/25 TRs) in steps of 1 TR, which resulting in 212 windows during rs-fMRI scanning. We chose the typical window size between 30 and 60 s, which was found to well balance the susceptibility to spurious fluctuations for short window lengths and categorical insensitivity to variability for long window lengths in empirical studies (Allen et al., 2014; Leonardi and Van De Ville, 2015; Zalesky and Breakspear, 2015). Following previous classic work, tapering window shape was suggested to better suppress spurious correlations and reduce sensitivity to outliers in categorically short time segments. The weighted Pearson correlation was adopted to calculate the FC for reducing the noise induced by the limited number of data points available in each time window (Zalesky et al., 2014). To estimate the dynamic FC within ventral subnetwork (dWNC), we computed the correlations between each pair of regions within the ventral subnetwork for each time window. Similarly, to characterize the dynamic FC between ventral and other subnetworks (dBNC), we extracted the correlations between each region in the ventral subnetwork and each region in the other subnetworks. Finally, all time windows were concatenated across all participants, resulting in 47700 (212 × 225) FC windows for dWNC and dBNC, respectively.

To detect the representative FC patterns for the dWNC, we applied the *k*-means clustering method on the concatenated FC matrix consisting of all participants’ time windows (Lloyd, 1982; Allen et al., 2014; Nomi et al., 2016). Following previous work in dynamics, the *k*-means algorithm was evaluated across values of *k* ranging from 2 to 10 using the silhouette metric, which measures how similar a FC window is to its own cluster compared to other clusters (Hutchison and Morton, 2015). The *k*-means clustering was repeated for 500 times with random initialization of centroid positions and produced the highest silhouette score with the value of *k* = 2. As a result, each time window for dWNC was assigned to one of the two typical dynamic FC states (clusters) for further analyses. Further, we calculated the frequency (i.e., the proportion of all 212 windows assigned to a particular state) and mean duration (i.e., the average number of consecutive windows assigned to a particular state) to describe each typical dynamic FC state. Likewise, we clustered the time windows for dBNC across all participants into two typical FC states and described their frequencies and mean durations in the same way.

### Behavioral correlation with dynamic functional connectivity states

The most essential nature of these typical dynamic states is their FC strength. Thus, we calculated the mean FC strength of each typical dynamic FC state for each participant, which was the averaged FC value for all windows assigned to a particular state. First, to examine how dynamic organization within the ventral subnetwork was related to representations of categorical and coordinate spatial relations, we conducted partial correlation analyses between the mean FC strength of each typical dWNC FC state and scores in the ordinal scene recognition test and the distance test, respectively. The same correlation analyses were performed between the mean FC strength of each typical dBNC FC state and two behavioral scores to examine how dynamic interactions between subnetworks supported representations of the two types of spatial relations. Several confounding factors were controlled. First, age and gender were included as control variables. Second, the framewise displacement (FD; *mean* = 0.09, *SD* = 0.03) (Power et al., 2012) was included as a control variable for head motion. Third, the familiarity of all landmarks was added as a control variable when performing the correlation analyses regarding the distance test.

## Results

**FIGURE 2:**
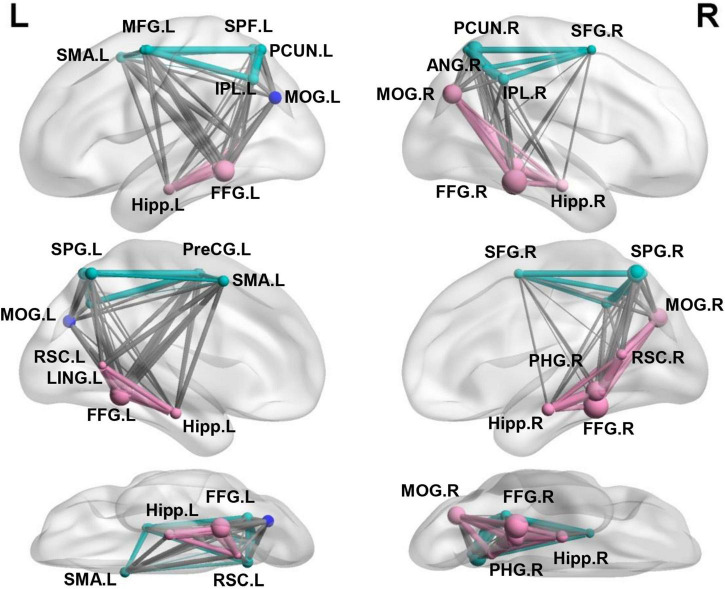
Separating the navigation network into the ventral and other two subnetworks. Three modules identified using modular analysis: ventral subnetwork (red) including most well-established regions for navigation and other two subnetworks (green and blue) including multiple regions in the dorsal parietal-frontal lobe.

In the present study, we used the Neurosynth-defined navigation network with 23 regions widely distributed in the medial temporal, parietal, and frontal cortex (Hao et al., 2021), which was aligned to another meta-analysis studies (Boccia et al., 2014, 2017; Ekstrom et al., 2017; Gona and Scarpazza, 2019). To refine the potential differences in navigation organization underlying between the representations of categorical and coordinate relations, we conducted a modularity analysis on the averaged FC matrix of the navigation network and obtained an optimized structure of three modules (Figure 2), with a modularity index (Q) of 0.50, indicating a strong modular structure in the navigation network (Newman and Girvan, 2004). The three modules almost fit well with three distinct pathways in the dorsal visual stream that mainly targets to the MTL involved in navigation, and projects to the prefrontal and premotro cortex involved in spatial working memory and visually guided action, respectively (Kravitz et al., 2011). Most important, the ventral subnetwork included bilateral HIP, parahippocampus gyrus (PHG), retrosplenial complex (RSC), lingual gyrus (LING), fusiform gyrus (FFG), and the right middle occipital gyrus (MOG), which were well-known navigational regions reported in previous studies (Epstein and Kanwisher, 1998; Maguire et al., 1998; Spiers and Maguire, 2006; Epstein, 2008; Epstein et al., 2017). Other subnetworks were widely characterized in many cognitions with general function, including bilateral superior parietal gyrus (SPG), Inferior parietal lobe (IPL), precuneus (PCUN), the right angular gyrus (ANG), superior frontal gyrus (SFG), and left precentral gyrus (PreCG), middle frontal gyrus (MFG), supplementary motor area (SMA), and a third module including the left MOG. Further examination found that the left MOG module consisted of multiple clusters, including some inferior parietal lobule voxels, a few superior parietal lobule voxels in Juelich Histological atlas and some superior lateral occipital cortex (LOC) voxels in Harvard-Oxford cortical structural atlas.

**FIGURE 3:**
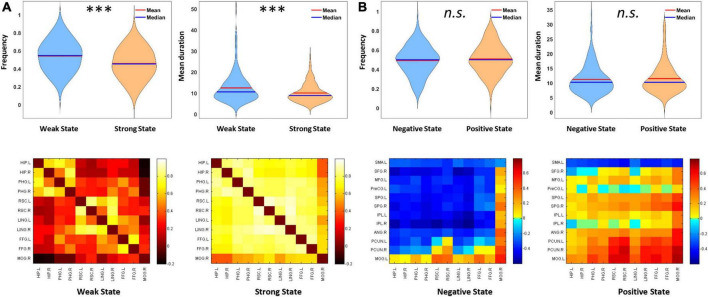
Properties of representative dynamic functional connectivity (FC) states within the ventral subnetwork (dWNC) and those between ventral and other subnetworks (dBNC). **(A)** Weak State and Strong State for dWNC. Weak State showed higher frequency, longer mean duration, and weaker FC strength than Strong State. **(B)** Negative State and Positive State for dBNC. The two states showed no difference in frequency or mean duration. Error bars indicate SEM. ***p < 0.001.

Next, we explored the representative states for dWNC using a clustering method. Stable clustering of all concatenated dWNC FC matrices was obtained, showing the highest silhouette value when *k* = 2. The resulting two clustering FC matrices represented the centroids of all matrices assigned to each cluster and putatively reflected the two typical FC states within the ventral subnetwork. As shown in Figure 3, we found that the two FC states showed different FC strength among the ventral regions, that is, one state showed relative weak FCs (mean = 0.28; *SD* = 0.051; named as Weak State), while the other showed strong FCs (mean = 0.62; *SD* = 0.05; named as Strong State; Weak vs. Strong, *t*_224_ = −101.65, *p* < 0.001). Notably, both the Weak and Strong State showed FC strength significantly higher than 0 (Weak State: *t*_224_ = 83.32, *p* < 0.001; Strong State: *t*_224_ = 185.94, *p* < 0.001), indicating functional integration within ventral subnetwork. Then, the Weak State had a higher frequency than the Strong State (Weak State: 0.54, Strong State: 0.46; *t*_224_ = 3.62, *p* < 0.001). Finally, the Weak State showed longer mean duration (12.34 ± 6.51 windows) than the Strong State (10.05 ± 4.36 windows; *t*_224_ = 3.85, *p* < 0.001).

Similarly, we adopted clustering analysis and revealed two typical states for all concatenated dBNC matrices. Specifically, one state showed significantly negative FCs between the ventral and other subnetworks (mean = −0.18, *SD* = 0.06; *t*_224_ = −48.48, *p* < 0.001; named as Negative State), indicating competitive interactions between subnetworks. In contrast, another state showed significantly positive FC (mean = 0.13, *SD* = 0.05; *t*_224_ = 36.47, *p* < 0.001; named as Positive State), indicating cooperative interactions between subnetworks. Apparently, two typical states showed significantly different mean FC strengths (*t*_224_ = −66.54, *p* < 0.001). There is no significant difference between the Negative and Positive States in frequency (Negative State: 0.49, Strong State: 0.51; *t*_224_ = −0.75, *p* = 0.45) or mean duration (Negative State: 11.22 ± 4.86 windows, Positive State: 11.51 ± 5.12 windows; *t*_224_ = −0.57, *p* = 0.57).

**FIGURE 4:**
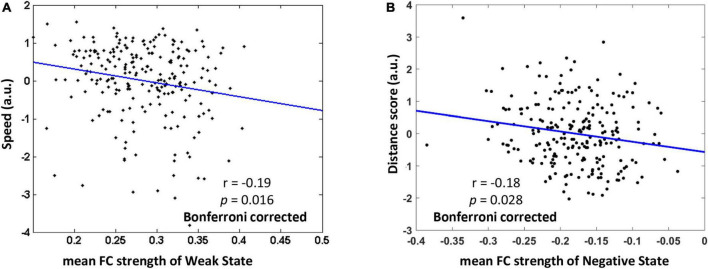
The relationship between mean functional connectivity (FC) strength of typical FC states and behavioral performance of representing categorial and coordinate spatial relations (Bonferroni correction). **(A)** Weaker mean FC of Weak State within the ventral subnetwork was related to higher speed in ordinal scene recognition based on representation of categorical spatial relations. **(B)** Stronger negative FC of Negative State between the ventral and other subnetworks was associated with higher distance scores based on representation of coordinate spatial relations. The speed score and distance score were standardized.

After depicting these typical states, we then explore how the mean FC strength of each typical dynamic FC state associates with behavioral performance of categorical and coordinate spatial relations. The descriptive statistics of behavioral tests were summarized in Table 1. There was no correlation between the speed scores and distance scores (*r* = −0.09, *p* = 0.20). On the one hand, for the dWNC, we examined how Weak State or Strong State were relevant to individual differences in representation of categorical spatial relations. To do this, we assessed the participants’ representation of categorical spatial relations by using an ordinal scene recognition task. We found the mean FC strength of the Weak State, not the Strong State, had a significant negative correlation with speed scores (0.71 ± 0.18), after controlling for age, gender, head motion (Weak State: *r* = −0.19, *p* = 0.016, Bonferroni correction; Strong State: *r* = −0.05, *p* = 0.42; Figure 4). These results suggested that weak integration among the ventral regions during resting-state was an optimal state for representation of categorical spatial relations. Next, we checked the association between dWNC FC states and participants’ ability to represent coordinate spatial relations. We didn’t find any correlations between distance scores and mean FC strength of the Weak State or Strong State (Weak State: *r* = 0.02, *p* = 0.78; Strong State: *r* = 0.02, *p* = 0.76). To sum up, these results confirm hypothesize 1, that is, representation of categorical spatial relations was related to weak dynamic integration within the ventral subnetwork.

**TABLE 1:** Demographic information and descriptive statistics of behavioral tests.

*n*		226
Sex (M/F)		108/118
Age		21.66 ± 1.00
**Categorical spatial relations**		
Ordinal scene recognition task	RT (s)	2.84 ± 1.077
	Speed	0.71 ± 0.183
	ACC	0.79 ± 0.136
**Coordinate spatial relations**		
Sense of distance score		5.28 ± 0.925
Path distance		6.19 ± 1.564
Euclidean distance		4.37 ± 2.139
Familiarity		6.10 ± 0.704

On the other hand, we investigated whether the FC states of dBNC were associated with the representation of coordinate spatial relations. We found that the mean FC strength of the Negative State, rather than the Positive State, was negatively correlated with the distance scores (5.28 ± 0.93), after controlling for age, gender, head motion, familiarity with landmarks (Negative State: *r* = −0.18, *p* = 0.028, Bonferroni correction; Positive State: *r* = −0.11, *p* = 0.11). These results suggested that stronger competitive interaction between the ventral and other subnetworks during resting-state was an optimal state for representation of coordinate spatial relations. Further, we checked the relationship between dBNC states and the representation of categorical spatial relations. We didn’t find the mean FC strength of the Negative State or Positive State had correlation with speed scores (Negative State: *r* = 0.14, *p* = 0.16; Positive State: *r* = 0.06, *p* = 0.35). In sum, these results confirmed hypothesize 2, that is, representation of coordinate spatial relations was related to dynamic interactions between the ventral and other subnetworks.

## Discussion

In the current study, we investigated the dissociable dynamic FC states underlying the representations of categorical and coordinate spatial relations in the large-scale organization of navigation network. First, we separated the navigation network into a ventral subnetwork containing the MTL regions and other two subnetworks with a modularity analysis. Then, we identified the Weak State and Strong State for dynamic FC within the ventral subnetwork and found that the Weak State was related to the performance of ordinal scene recognition based on categorical relations, suggesting that the representation of categorical spatial relations was related to weak integration among focal regions within the ventral subnetwork. In contrast, we identified the Negative State and Positive State for dynamic FC between the ventral and other subnetworks and found that the Negative State was associated with the distance test scores assessing coordinate relations, suggesting that the representation of coordinate spatial relations may require competitive interactions among widely distributed regions between the ventral and dorsal subnetworks. Overall, our study provides the first empirical evidence at the network level revealing dissociation of focal and distributed organizations of the navigation network in representing different types of spatial information, which may illuminate the mechanisms for understanding scenes containing multiple objects.

Importantly, our study revealed dynamic organizations of the navigation network during resting-state. First, we revealed two opposite FC states for interactions between the ventral and other two subnetworks, a positive state and a negative state. Previous studies using static FC methods reported only positive connectivity between the ventral and dorsal networks. For instance, the RSC was functionally connected with widespread parieto-frontal regions including the posterior cingulate cortex, the PCUN, and SFG (Boccia et al., 2017). Intriguingly, for the first time we revealed that FCs between the ventral and other subnetworks alternated between a positive and a negative state, suggesting that cooperative interactions between subnetworks are accompanied by periods of competitive interactions between them during resting-state. While the positive state may promote efficient communication between the subnetworks, the negative state may constrain the information flow between them, contributing to the functional specialization of the ventral subnetwork. Additionally, we revealed a weak state and a strong state for FCs within the ventral subnetwork. Previous findings have reported wide-spread positive resting-state FCs among ventral subnetwork regions, such as the HIP, parahippocampal place area, RSC, and occipital place area (Kong et al., 2016; Silson et al., 2016; Boccia et al., 2017). Our results enriched previous findings by showing that integration within the ventral subnetwork also alternated between two typical states with different FC strength, a strong state and a weak state, each potentially exploited to varying degrees by navigational behaviors. While the strong state indicated highly synchronized activity among the ventral subnetwork regions, the weak state exhibited loose synchronization activity among the regions.

Further, identification of different dynamic FC states is critical to reveal the association between spatial relation representations and organizations of the navigation network, since our results indicated that spatial relation representations were associated with FC states of only some but not all time periods during resting-state. Specifically, representation of categorical spatial relations was only associated with the Weak State, but not the Strong State, within the ventral subnetwork; in contrast, representation of coordinate spatial relations was only related to the Negative State, but not the Positive State, between the ventral and other subnetworks. The associations between spatial relationship representations and specific dynamic FC states were concealed when FCs of all time windows were taken as static and invariant.

The dissociation of representations of categorical and coordinate spatial relations lies in two aspects. First, representation of categorical spatial relations was mainly related to the ventral subnetwork, while representation of coordinate spatial relations was related to interactions between the ventral and other subnetworks. Although some studies have suggested the potential dissociable neural bases between the hippocampal formation and parietal cortex underlying representations of the two types of spatial relations, accumulating research showed different results and indicated the dissimilarity might didn’t be confined to the specific regions. Broad regions were found be involved in the two kinds of spatial relations. The MTL has been found to play a central role in representing both categorical and coordinate spatial relations (Howard et al., 2014; Blacker and Courtney, 2016; Deuker et al., 2016; Urgolites et al., 2017), and the prefrontal and posterior parietal regions are also involved in coordinate spatial relations (Spiers and Maguire, 2007; Morgan et al., 2011; Viard et al., 2011). Our results extended previous studies by comparing the dynamic FC nature of the two types of spatial relations from the network organization level. Representation of categorical spatial relations has been considered as an integrated cognitive process including extracting environment layout in the PPA, encoding location information in the HIP, and updating viewpoint information in the RSC. It’s reasonable that the dynamic cooperation of ventral navigation regions supports the representation of categorical spatial relations. In contrast, representation of coordinate spatial relations requires precise distance information between landmarks, possibly involving both spatial processing and high-level cognitive functions such as executive control and attention modulation, which may be supported by dynamic communication between the ventral and dorsal subnetworks.

Another dissociation of representations of the two types of spatial relations lies in that weak integration among the ventral regions during resting-state was optimal for representation of categorical spatial relations, while competitive interaction between the ventral and other subnetworks was optimal for representation of coordinate spatial relations. It can be speculated that the moderate integration among the ventral regions may support an optimal balance between effective communications among these regions and maintenance of independent function of individual regions. In contrast, the competitive interactions between the ventral and other subnetworks are important for precise representation of coordinate spatial relations. In line with this result, we have found in a previous study that stronger integration of the IPS with other regions in the navigation network was associated with poor ability of executive control (Hao et al., 2017). Thus, we speculated that competitive interactions between the ventral and parietal-frontal subnetworks might be related to better ability of executive control or other high-level cognitive functions, which brings better representation of coordinate spatial relations.

Our study revealed different dynamic network organization states in relation to the representations of categorical and coordinate spatial relations. It is worth noting that the distinction of allocentric and egocentric spatial processing constitutes another vital classification, which concerns about the frame of reference with respect to navigator or environment. The allocentric-egocentric dichotomy may partly overlap with the categorical-coordinate dichotomy. It will be still necessary for future studies to further determine the relationship between the two dichotomy systems (Jager and Postma, 2003; Baumann and Mattingley, 2014; Ruotolo et al., 2019). Several important issues are unaddressed for future research. First, the present study characterized dynamic organizations of the navigation network by clustering FC states of all time windows into typical FC states, and future study needs to examine how more quantitative characteristics in dynamic network organization, such as flexibility and module allegiance, are related to spatial relation representation (Chai et al., 2016). Noteworthy, although we didn’t find any sub-modules for the ventral network in the weak state, it still inspires further studies to explore the functional significance of possible sub-modules in the ventral network with more sensitive measures. Second, Deco et al. (2011) propose that dynamic network configuration is constrained by the underlying stable anatomical skeleton, and it’s important to explore the link between anatomical structure and resting-state functional dynamics. Third, the negative correlation between the ventral and dorsal subnetworks should be interpreted with caution, considering the debate that global signal regression may introduce artifactual anti-correlations (Fox et al., 2005; Murphy et al., 2009; Cole et al., 2010; Murphy and Fox, 2017). Forth, the role of MOG module seems to be special. Further research would be valuable to examine the temporal variability at the nodal level and subnetwork level in an integrated manner (Zhang et al., 2016; Sun et al., 2019), which helps to better reveal the functional specificity of navigation network regions. Finally, the dynamic FC patterns revealed in our study provide new insights than constant connectivity patterns in conventional analysis, and future studies with the dynamic approach in developing brain may provide new understanding of brain maturation and plasticity.

## Data availability statement

The raw data supporting the conclusions of this article will be made available by the authors, without undue reservation.

## Ethics statement

The studies involving human participants were reviewed and approved by Institutional Review Board of Beijing Normal University. The patients/participants provided their written informed consent to participate in this study.

## Author contributions

XH and YS designed the experiments and wrote the manuscript. XH, ZC, and XK conducted the experiments. XH and TH analyzed the data. XH, YS, and JL supervised the project. All authors contributed to the article and approved the submitted version.

## Footnotes

1
http://www.fmrib.ox.ac.uk/fsl
